# Supplementary material for: Evaluation of Avian Reovirus S1133 Vaccine Strain in Neonatal Broiler Chickens in Gastrointestinal Integrity and Performance in a Large-Scale Commercial Field Trial
Source: Vaccines (Basel). 2021 Jul 23;9(8):817. doi: 10.3390/vaccines9080817 (PMC8402455; doi:10.3390/vaccines9080817)
Supplement: Supplementary file 1 [file vaccines-09-00817-s001.zip › vaccines-1270430-supplementary.pdf]

Table S1. Ingredient composition (kg) and nutrient content of the phases of feed supplied to the experimental female broiler chickens.

Ingredients	Pre-starter 1–7 days	Starter 8–20 days	Grower 21–30 days	Finisher 31 days to harvest
White corn 8.00%			676	694
Yellow corn 7.83%	559.5	613.5		
Soybean meal 46.5%	385	335	273.5	258.5
Vegetable oil	16	16	17	17
Limestone 38% Ca	7.219	7.585	5.125	4.235
Mono-di calcium phosphate 21% P, 18% Ca	11.0	8.5	7.0	6.0
Aluminosilicate			3.0	3.0
Liquid L-lysine 50% (ADM, Chicago, IL, USA)	3.500	3.300	3.900	3.800
Methionine hydroxy analog 88% (ALIMET 88®, Novus International, Saint Charles, MO, USA)	3.700	3.300	2.800	2.800
NaCl (refined salt)	3.900	3.500	3.100	3.100
Sodium bicarbonate	1.300	1.300	1.500	1.500
Vitamin premix (Chicken AQ V1, Trouw Nutrition, Zapopan, Jal. Mexico)	1.200	1.000	1.000	0.850
Organic Zinc and Manganese (Availa®Z/M, Zinpro Corp., Eden Prairie, MN, USA)	1.0	1.0		
Organic aluminosilicate (Zeotek® (Sanfer, Alvaro Obregon, Mexico City, Mexico)	1.0	0.8	0.50	0.50
Carboxylic acid premix (Sal Plus Ultra Polvo®, CFS Dresen, Urbandale, IA, USA)	1.0	1.0	1.0	1.0
Sodium butyrate 92% (Gustor B-92® Norel, Madrid - España)	1.0			
Mineral premix [19]	0.800	0.800	0.800	0.800
L-Threonine (ThreAMINO®, Evonik, Essen, Germany)	0.750	0.800	1.000	0.800
β-Mannanase (CIBENZA DE200® Novus International, Inc., St. Louis, MO, USA)	0.500	0.500	0.500	0.500
Salinomicin 12% (Coxistac® 12%, Phibro Animal Health Corp., Teaneck, NJ, USA)			0.600	0.00
Biocholine Conjugated (BioCholine Powder®, Nuproxa, LTD, Switzerland)	0.666	0.500	0.460	0.400
Nicarbazin 25% (Avicarb®, Phibro Animal Health Corp., Teaneck, NJ, USA)		0.650		
Lithic cycle bacteriophages (eXolution® Bacteriophage F. CTCBio®, Seoul, South Korea)	0.500	0.500	0.500	0.500
Organic Zinc (Availa®Zn 120, Zinpro Corporation, Eden Prairie, MN, USA)			0.25	0.25
Protease (Poultry Grow 250®, Jefo Nutrition Inc., Saint-Hyacinthe, Canada)	0.125	0.125	0.125	0.125
Colistin sulfate 4% (Colimix® virbac, Westlake, TX, USA)	0.250	0.250	0.250	0.250
6-Phytase (Axtra PHY TPT 10000®, Dupont Industrial Biosciences, Marlborough, UK)	0.050	0.050	0.050	0.050
Virginiamycin 50% (Stafac® 500, Phibro Animal Health Corp., Teaneck, NJ, USA)	0.040	0.040	0.040	0.040
**Nutrients**				
Weight	1.0	1.0	1.0	1.0
Dry matter (%)	88.30	88.35	88.52	88.50
Crude protein (%)	23.000	20.900	19.000	18.500
Total lysine (%)	1.450	1.300	1.170	1.130
Methionine total (%)	0.660	0.600	0.540	0.530
Methionine + cystine (%)	1.040	0.960	0.880	0.860
Total tryptophan (%)	0.285	0.256	0.215	0.206
Threonine total (%)	0.945	0.880	0.805	0.767
Metabolizable energy (Mcal kg^{-1})	3.040	3.100	3.210	3.230
Crude fat (%)	4.300	4.500	5.600	5.600
Total calcium (%)	0.950	0.900	0.800	0.700
Total phosphorus (%)	0.61	0.55	0.50	0.47
Phosphorus available (%)	0.500	0.450	0.400	0.390
Total chlorine (%)	0.260	0.240	0.220	0.220
Total sodium (%)	0.200	0.185	0.180	0.180
Total potassium (%)	1.000	0.942	0.735	0.742
Mineral materials (%)	5.80	5.60	5.35	5.22
Linoleic acid (%)	2.20	2.25	2.44	2.57
Crude fiber (%)	2.60	2.60	2.50	2.49

Table S2. Feeding phase costs and cost breakdown of experimental female broilers.

**Feed cost**	**%**	**USD·chick^{-1}**	**Pre-starter** 1–7 days	**Starter** 8–20 days	**Grower** 21–30 days	**Finisher** 31 days to harvest
Cost by feed phase (USD·1,000 kg^{-1})			$495.00	$485.15	$449.87	$445.17
Consumption per chicken per feed pase (g)			190	810	1,000	705
Cost breakdown						
Feed	61					
Chick	16					
Marketing, sales, and distribution	7					
Labor	6					
Electricity, water, and gas	6					
Medication	2					
Other	2					
Total	100					
Reovirus vaccine to S1133ARV group		$0.0102				
